# Immediate thoracotomy for penetrating injuries: ten years’ experience at a Dutch level I trauma center

**DOI:** 10.1007/s00068-012-0198-6

**Published:** 2012-06-16

**Authors:** O. J. F. Van Waes, P. A. Van Riet, E. M. M. Van Lieshout, D. D. Hartog

**Affiliations:** Department of Surgery-Traumatology, Erasmus MC, University Medical Center Rotterdam, Room H-822k, P.O. Box 2040, 3000 CA Rotterdam, The Netherlands

**Keywords:** Thoracic trauma

## Abstract

**Background:**

An emergency department thoracotomy (EDT) or an emergency thoracotomy (ET) in the operating theater are both beneficial in selected patients following thoracic penetrating injuries. Since outcome-descriptive European studies are lacking, the aim of this retrospective study was to evaluate ten years of experience at a Dutch level I trauma center.

**Method:**

Data on patients who underwent an immediate thoracotomy after sustaining a penetrating thoracic injury between October 2000 and January 2011 were collected from the trauma registry and hospital files. Descriptive and univariate analyses were performed.

**Results:**

Among 56 patients, 12 underwent an EDT and 44 an ET. Forty-six patients sustained one or multiple stab wounds, versus ten with one or multiple gunshot wounds. Patients who had undergone an EDT had a lower GCS (*p* < 0.001), lower pre-hospital RTS and hospital triage RTS (*p* < 0.001 and *p* = 0.009, respectively), and a lower SBP (*p* = 0.038). A witnessed loss of signs of life generally occurred in EDT patients and was accompanied by 100 % mortality. Survival following EDT was 25 %, which was significantly lower than in the ET group (75 %; *p* = 0.002). Survivors had lower ISS (*p* = 0.011), lower rates of pre-hospital (*p* = 0.031) and hospital (*p* = 0.003) hemodynamic instability, and a lower prevalence of concomitant abdominal injury (*p* = 0.002).

**Conclusion:**

The overall survival rate in our study was 64 %. The outcome of immediate thoracotomy performed in this level I trauma center was similar to those obtained in high-incidence regions like the US and South Africa. This suggests that trauma units where immediate thoracotomies are not part of the daily routine can achieve similar results, if properly trained.

## Introduction

Thoracic injuries represent one of the leading causes of death in all age groups, and account for 25–50 % of all traumatic injuries [1]. Thoracic trauma ranks third, after head and extremity trauma, among major accidents in the United States (US), and is responsible for approximately half of all traumatic deaths [2]. Most penetrating injuries of the chest can be managed nonoperatively or with minimally invasive techniques. A small but significant group of 10–15 % of patients with penetrating thoracic injuries require an immediate thoracotomy as part of their initial resuscitation. An immediate thoracotomy can be performed in the operating theater, herein referred to as an “emergency thoracotomy” (ET), or at the emergency department (ED), herein referred to as an “emergency department thoracotomy” (EDT). Survival rates after an immediate thoracotomy following penetrating thoracic trauma are usually reported to be around 9–12 % [3], but have been reported to be as high as 38 % [4]. Much effort has been devoted to identifying patients who are likely to benefit from an immediate thoracotomy [5–9]. Most of the experience of performing immediate thoracotomies has been gained in high-incidence regions like the US and South Africa [7, 8]. Although penetrating trauma accounts for only 5–10 % of all trauma in Europe, compared with 40–50 % in the US, the incidence rates of patients presenting to an ED in the Netherlands with penetrating injury has gradually increased over the past few years, by up to 8 % annually [10]. Despite this rise in incidence in the Netherlands and other European countries, there is a paucity of studies from Europe regarding the use and outcome of an immediate thoracotomy following penetrating thoracic trauma. Moreover, outcome-related physiologic parameters have only been validated in three studies [11–13], which makes it even more difficult to interpret and use these data in the European emergency situation [3].

Ten years ago, immediate thoracotomy in the management of life-threatening thoracic penetrating injury was embedded in our level I trauma center. Since the experience of performing immediate thoracotomies in Europe is limited compared with the US and South Africa [14, 15], the aim of this study was to evaluate our ten years of experience with immediate thoracotomy and to describe the practices and outcomes of penetrating thoracic trauma.

## Methods

### Study setting

This study was performed at a level I trauma center in the southwestern part of the Netherlands. This 1300+ bed university medical center serves a population of 4.9 million. Patients who have sustained penetrating chest injuries in our adherence area are announced by pre-hospital care providers (either ambulance or helicopter emergency medical services), after which a trauma team is assembled (available 24/7). The team consists of a trauma surgeon (head of the trauma team), a surgical resident, an anesthesiologist, an emergency physician, two emergency nurses, and a radiologist. Blood products and surgical equipment for either thoracotomy or sternotomy are available in the resuscitation room. In case of a resuscitative EDT, both the thoracic surgeon and the operating theater facilities are notified for subsequent definitive care. In hemodynamically stable patients, computed tomographic angiography (CTA) is readily available opposite to the resuscitation room if required.

### Patient selection

Patients who underwent an immediate thoracotomy after sustaining penetrating thoracic injury between October 2000 and January 2011 were selected from the trauma registry. An immediate thoracotomy was defined as a thoracotomy required as an integral part of the initial resuscitation of the trauma patient in the ED, or for imminent surgical repair of the injuries in the operating theater [16]. Both ET and EDT were included. An ET was performed in resuscitation-responsive patients (systolic blood pressure (SBP) ≥60 mmHg), versus an EDT in resuscitation-unresponsive or transient patients with a SBP <60 mmHg. Both thoracotomies allow the evacuation of pericardial tamponade, direct control of intrathoracic hemorrhage, control of massive air embolism, open cardiac massage, and cross-clamping of the descending aorta to redistribute blood flow and limit subdiaphragmatic hemorrhage [17, 18]. Patients who had only undergone an elective thoracotomy were excluded. An elective thoracotomy was defined as a procedure to correct nonacute life-threatening thoracic injury or postinjury complications such as empyema. Patients receiving a thoracotomy after blunt thoracic trauma or after a nontraumatic thoracic injury (indicated when massive intrathoracic or abdominal bleeding occurs) were also excluded.

### Intervention

**Fig. 1:**
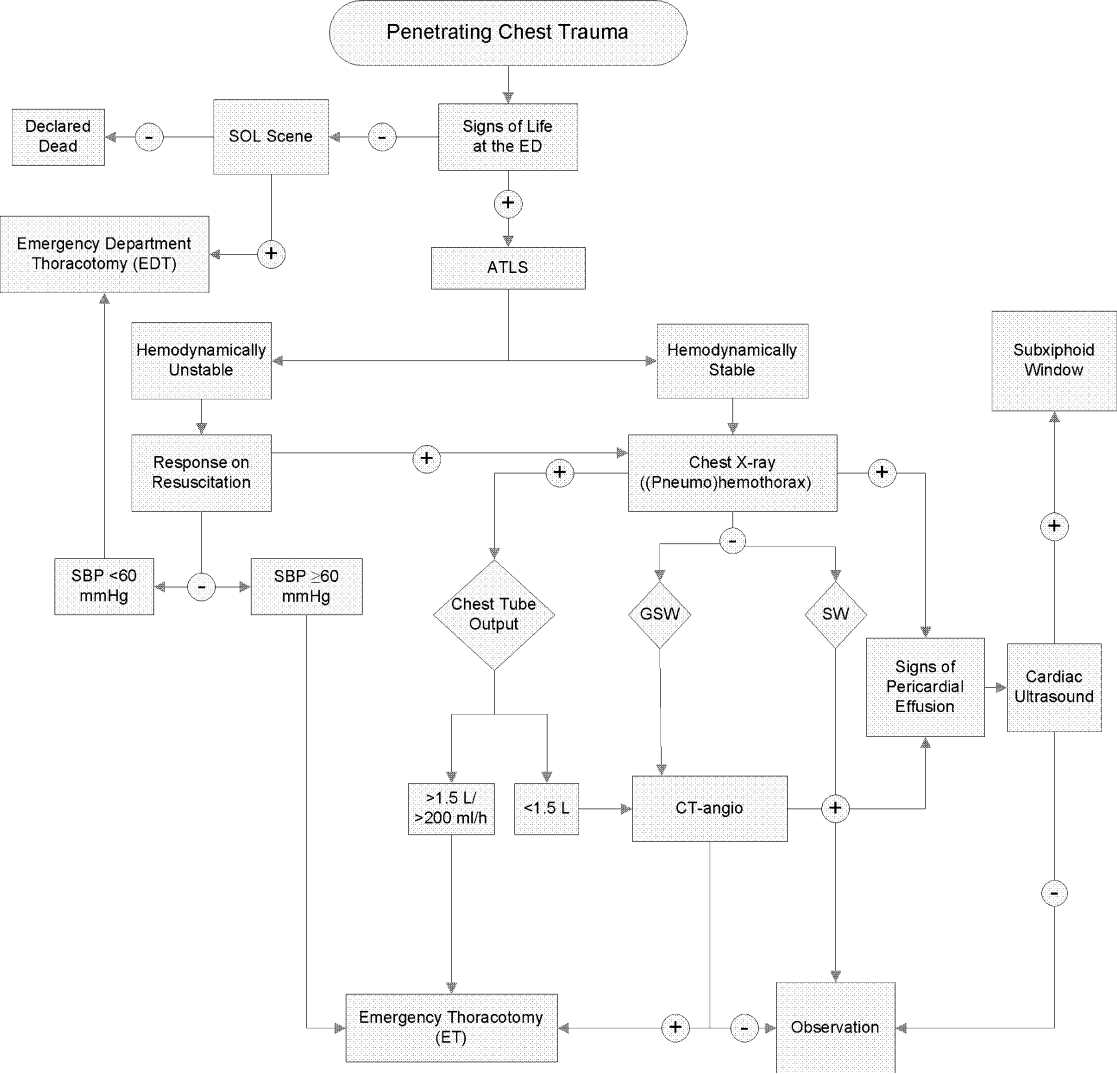
Flowchart with decision-making pathway for an immediate thoracotomy after penetrating chest trauma. *ATLS* advanced trauma life support, *ED* emergency department, *SOL* signs of life, *SBP* systolic blood pressure, *GSW* gunshot wound, *SW* stab wound, *CT-angio* computed tomography angiography. A hemodynamically unstable condition was defined as a SBP <100 mmHg with or without a response to resuscitation. A hemodynamically stable condition was defined as an SBP of ≥100 mmHg

**Fig. 2:**
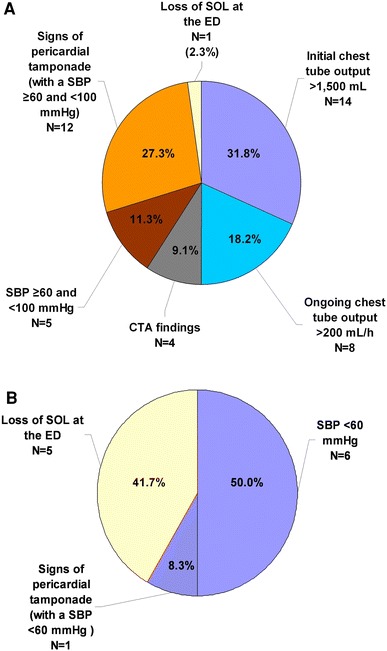
Indications for performing an ET (**a**) or an EDT (**b**). *SOL* signs of life, *ED* emergency department. Persisting shock was defined as a systolic blood pressure of ≥60 and <100 mmHg and no response to resuscitation or a transient response. Severe shock was defined as a systolic blood pressure of <60 mmHg and no response to resuscitation or a transient response. CTA findings included gross contrast extravasation, a hemothorax, or air leakage

Advanced trauma life support (ATLS) guidelines were used for initial assessment and treatment [19]. Patients who sustained penetrating thoracic injuries were managed as shown in Fig. 1. Indications for an EDT and an ET are shown in Fig. 2. Indications for an EDT included (1) loss of signs of life (SOL) on arrival at the ED but presence of SOL at the scene of injury, and (2) failure to respond to resuscitation with a SBP <60 mmHg. Pericardial tamponade only represented an indication for an EDT when accompanied with an associated SBP <60 mmHg. ET indications included (1) a hemothorax on chest X-ray (CXR) with an initial chest tube output of >1,500 mL or an ongoining chest tube output of >200 mL/h for 2–4 h after insertion of the tube, (2) a hemothorax on CXR with a chest tube output of <1,500 mL, but with CTA of the chest findings prompting surgical intervention (e.g., gross contrast extravasation or air leakage), (3) signs of pericardial tamponade, or (4) a massive air embolism [19]. Operative maneuvers performed during a thoracotomy and/or a laparotomy are shown in Table 2. Table 2 shows the operative findings following a thoracotomy and/or an additional laparotomy.

### Data collection

Data on patient characteristics, injury characteristics, physiological parameters, and outcome were prospectively collected in and retrieved from our trauma registry and the patient hospital files. Data collected included age, gender, mechanism of injury, SOL, Glasgow coma scale (GCS score), injury severity score (ISS) [20], triage revised trauma score (triage RTS) [21], SBP, the need for cardiopulmonary resuscitation (CPR), transportation time, indications for thoracotomy, operative maneuvers, intraoperative findings, and complications. The length of hospital stay (H-LOS) was categorized as <24 or >24 h. Presence of SOL was defined by at the presence of at least one of the following: GCS >3, respiratory effort, cardiac activity on ECG or ultrasound (with or without a pulse), or evidence of pupillary reflexes. ISS was scored according to the abbreviated injury scale (AIS-90) [22]. CPR was performed according to the guidelines for resuscitation of the European Resuscitation Council (2005) [23].

### Data analysis

Statistical analysis was performed using the Statistical Package for the Social Sciences (SPSS) v.16.0 (SPSS, Chicago, IL, USA). Continuous data were tested for normality with the Shapiro–Wilk and Kolmogorov–Smirnov tests, and by inspecting the frequency distributions (histograms). Homogeneity of variance was checked for using Levene’s test. Since most of the continuous data were skewed, all data were analyzed using a nonparametric Mann–Whitney *U* test. Categorical data were compared using Fisher’s exact test or a chi-squared test: in small samples, or if the assumptions of the chi-squared test were not met, Fisher’s exact test was performed. *P* values of <0.05 were considered statistically significant.

## Results

Over a ten-year period, a total of 416 patients with penetrating thoracic injury were referred to the ED; 72 presented with one or more gunshot wounds, and 344 with one or more stab wounds. Among all 416 patients, 346 patients presented only with thoracic trauma, while 70 patients presented with both thoracic and abdominal trauma. An intervention was indicated in 127 of 416 patients, including 39 thoracotomies, 32 laparotomies, and 17 patients who underwent both a thoracotomy and a laparotomy. The remaining 39 patients underwent other operative interventions. Among all 56 patients who underwent an immediate thoracotomy, 46 patients sustained a stab wound and 10 patients a gunshot wound. The male to female ratio was 6:1, and the median age was 32 years (*P*_25_–*P*_75_ 25–41 years).

Among the 56 patients included in this study, 12 underwent an EDT and 44 an ET. Demographic and physiological data on these patients are shown in Table 1. In terms of the mechanism of injury, more gunshot wounds were found in the EDT group than in the ET group (*p* = 0.028). Overall, stab wounds dominated in both groups. Patients in the EDT group had a lower pre-hospital GCS (*p* < 0.001), lower pre-hospital RTS and hospital triage RTS (*p* < 0.001 and *p* = 0.009, respectively), and a lower hospital SBP (*p* = 0.038) than patients in the ET group. ISS, however, was similar in both groups.

**Table 1:** Patient characteristics of the study population in whom immediate thoracotomy was performed in the ED (EDT) or in the operating theater (ET)

Parameter	Overall (*n* = 56)	EDT (*n* = 12)	ET (*n* = 44)	*p* value
Pre-hospital				
Age (years)^a^	32 (25–41)	28 (24–41)	33 (25–41)	0.555^c^
Gender (men)^b^	48 (86)	10 (83)	38 (86)	N.S.^c^
Stab wounds^b^	46 (82)	7 (58)	39 (89)	0.028^d^
Signs of life^b^	55 (98)	12 (100)	43 (98)	N.S.^c^
Glasgow coma score^a^	14 (3–15)	3 (3–10)	14 (12–15)	<0.001^c^
Systolic blood pressure (mmHg)^a^	98 (60–114)	0 (0–110)	100 (80–120)	0.0140^c^
Revised trauma score^a^	11.00 (7.00–12.00)	4.50 (4.00–7.00)	12.00 (8.50–12.00)	<0.001^c^
Closed-chest cardiopulmonary resuscitation^a^	6 (11)	0 (0)	6 (14)	N.S.^e^
In-hospital				
Time until ED arrival (min)^a^	24 (15–32)	13 (2–23)	33 (18–35)	0.006^c^
Time until thoracotomy (min)^a^	68 (42–128)	25 (15–107)	79 (52–155)	0.037^c^
Cardiopulmonary resuscitation^b^	17 (30)	9 (75)	8 (18)	<0.001^d^
Signs of life^b^	50 (89)	7 (58)	43 (98)	0.001^c^
Systolic blood pressure (mmHg)^a^	105 (69–120)	0 (0–113)	107 (80–126)	0.038^c^
Injury severity score^a^	25 (16–34)	34 (17–36)	20 (15–34)	N.S.^c^
Triage-revised trauma score^a^	8 (4–8)	4 (1–8)	8 (5–8)	0.009^c^
H-Los (days)^a^	7 (0–12)	0 (0–5)	8 (5–14)	0.005^c^
IC-LOS (days)^a^	1 (0–3)	0 (0–2)	1 (1–3)	0.012^c^

^a^ Data are displayed as the median, with the first and third quartiles given in parentheses

^b^ Patient numbers are displayed, followed by the corresponding percentages in parentheses

^c^ Mann–Whitney *U* test, ^d^ Fisher’s exact test, ^e^ Chi-squared test

*H-LOS* hospital length of stay, *IC-LOS* duration of stay at the intensive care unit

Cardiopulmonary resuscitation was performed in 19 patients, of which six received pre-hospital closed chest cardiopulmonary resuscitation (CC-CPR). All six patients who received pre-hospital CC-CPR, with or without additional in-hospital CPR, progressed to an ET. Of these six patients, five received an EDT before they were transported to the operation room for an EDT. The majority of the patients receiving in-hospital CPR underwent an EDT (*p* < 0.001). The median time interval from the arrival of emergency medical services at the scene of injury until admittance to the ED was shorter in the EDT group (13 min;* P*_25_–*P*_75_ 2–23) than in the ET group (33 min;* P*_25_–*P*_75_ 18–35; *p* = 0.006). The median time span from injury scene to thoracotomy was also shorter in the EDT group (25 min;* P*_25_–*P*_75_ 15–107) than in the ET group (79 min;* P*_25_–*P*_75_ 52–155; *p* = 0.037; Table 1).

Among all 56 immediate thoracotomies, ten were performed within 1 h after injury, 14 within 1–3 h, and six within 4–10 h. The transportation times of 26 patients could not be obtained. The indications for an ET are presented in Fig. 2a, and the indications for an EDT are shown in Fig. 2b. Indications are in agreement with the flowchart in Fig. 1.

A total of 64 incisions were performed: 22 midsternal incisions, 20 left anterolateral, ten right anterolateral, two left posterolateral, six right posterolateral, and four clamshell. Operative findings and maneuvers for EDT and ET are shown in Table 2. Hemothorax was found significantly more often in the ET group. Internal cardiac massage and pulmonary hilar twist were performed more frequently in the EDT group (*p* < 0.001 and *p* = 0.043, respectively). Abdominal trauma was found in ten of all 17 patients undergoing an additional laparotomy, and was not observed more often in either the ET or the EDT group (*p* = 0.433). The most common intra-abdominal findings were damage to the diaphragm and the liver.

**Table 2:** Operative findings (A) and maneuvers (B) during EDT versus ET

	Overall (*n* = 56)	EDT (*n* = 12)	ET (*n* = 44)	*p* value
(A) Operative findings (per patient)				
Hemothorax	41 (73)	6 (50)	35 (80)	0.039^a^
Lung injury	27 (48)	4 (33)	23 (52)	0.334^b^
Cardiac injury	28 (50)	7 (58)	21 (48)	0.746^b^
Diaphragm perforation	6 (11)	0 (0)	6 (14)	0.359^a^
Transection of intrathoracic vessels	8 (14)	4 (33)	9 (20)	0.055^b^
(B) Operative maneuvers (per patient)				
Control of intrathoracic hemorrhage	47 (84)	9 (75)	38 (86)	0.385^b^
Release of pericardial tamponade	16 (29)	4 (33)	12 (27)	0.726^b^
Internal cardiac massage	13 (23)	7 (58)	6 (14)	<0.001^b^
Pneumectomy	3 (5)	0 (0)	3 (7)	0.512^b^
Pulmonary hilar twist or clamp	2 (4)	2 (17)	0 (0)	0.043^b^
Wedge resection	2 (2)	0 (0)	2 (5)	N.S.^b^
Aortic cross-clamping	1 (2)	1 (8)	0 (0)	0.214^b^

Data are shown as numbers with the corresponding percentages between parentheses, and were analyzed using the^ a^ Chi-squared test or ^b^ Fisher’s exact test

In the survivors, postoperative complications occurred in 20 patients, of whom five experienced one or more complications (Table 3). Complications ranged from superficial wound infection to re-bleeding in six patients.

**Table 3:** Complications following EDT and ET

Complications	Overall (*n* = 56)	EDT (*n* = 12)	ET (*n* = 44)
Mortality	20 (36)	9 (75)	11 (25)
Re-bleeding	6 (11)	1 (8)	6 (14)
Acute respiratory distress syndrome	2 (4)	1 (8)	1 (2)
Superficial wound infection	1 (2)	0 (0)	1 (2)
Abscess	2 (4)	0 (0)	2 (5)
Pneumonia	3 (5)	1 (8)	2 (5)
Empyema	2 (4)	0 (0)	2 (5)
Sepsis	1 (2)	0 (0)	1 (2)
Rhabdomyolysis	2 (4)	1 (8)	1 (2)
Neurological impairment	2 (4)	0 (0)	2 (5)
Re-operation	9 (16)	1 (8)	8 (18)

Data are shown as numbers with the corresponding percentages between parentheses

Complications other than mortality are shown for survivors only

Re-operation was performed in nine patients and included two laparotomies and seven re-thoracotomies. Among this latter group, two patients underwent an elective thoracotomy and five a re-thoracotomy due to persistent thoracic blood loss. Operative findings following persistent thoracic blood loss included progressive rupture of the cardiac apex despite the placement of several cardiac sutures 2 h earlier, continuous bleeding of intercostal vessels, laceration of the aortic arch, bleeding of the subclavian artery, and a negative re-thoracotomy in one patient. The overall survival of patients was 64 %: 25 % in the EDT group and 75 % in the ET group (Table 4). In the EDT group, five out of 12 patients (42 %) advanced to definitive surgical care. The three patients who survived an EDT left the hospital without neurological impairment. Among all 44 patients in the ET group, 33 (75 %) survived until discharge, of whom 31 (94 %) were neurologically intact.

**Table 4:** Factors associated with mortality after an immediate thoracotomy

Factors	Total (*n* = 56)	Nonsurvivors (*n* = 20)	Survivors (*n* = 36)	*p* value
Pre-hospital				
Signs of life^b^	55 (98)	19 (95)	36 (100)	0.357^d^
Pupillary response^b^	45 (80)	11 (55)	34 (94)	0.002^e^
Triage-revised trauma score^a^	11 (7–12)	8 (4–11)	12 (10–12)	0.001^c^
Glasgow coma scale^a^	14 (3–15)	3 (3–13)	15 (13–15)	<0.001^c^
Systolic blood pressure (mmHg)^a^	98 (60–114)	68 (0–109)	101 (80–127)	0.009^c^
Hemodynamic unstable^b^	29 (52)	15 (75)	14 (39)	0.031^e^
Gunshot wound^b^	10 (17)	6 (30)	4 (11)	0.142^d^
Abdominal injury^b^	10 (18)	8 (40)	2 (6)	0.002^d^
In-hospital				
Injury severity score^a^	25 (16–34)	34 (17–45)	20 (12–30)	0.011^c^
Triage-revised trauma score^a^	8 (4–8)	4 (1–8)	8 (6–8)	0.008^c^
Systolic blood pressure (mmHg)^a^	105 (69–120)	70 (0–108)	110 (91–130)	0.003^c^
Signs of life^b^	50 (89)	14 (70)	36 (100)	0.001^d^
CPR^b^	17 (30)	15 (75)	2 (6)	<0.001^d^
EDT^b^	12 (21)	9 (45)	3 (8)	0.002^d^
Transection of intrathoracic vessels^b^	8 (14)	6 (30)	2 (6)	0.019^d^
Thoracotomy indications				0.003^e^
Pericardial tamponade^b^ (with associated shock)	13 (23)	2 (10)	11 (31)	
Ongoing chest tube production >200 mL/h^b^	8 (14)	1 (5)	7 (19)	
Hemodynamically unstable condition^b^	11 (20)	7 (35)	4 (11)	
Absence of signs of life^b^	5 (9)	5 (25)	0 (0)	

^a^Data are displayed as the median, with the first and third quartiles given within parentheses

^b^Patient numbers are displayed, with the percentages given within parentheses

Data were analyzed using ^ c^ The Mann–Whitney *U* test, ^d^ Fisher’s exact test, ^e^ The chi-squared test

*ED* emergency department, *CPR* cardiopulmonary resuscitation, *EDT* emergency department thoracotomy. A pre-hospital hemodynamically unstable condition was defined as an SBP of <100 mmHg or no response to resuscitation. A hemodynamically unstable condition as an indication for thoracotomy was defined as an SBP of <60 mmHg or no response to resuscitation

The physiological conditions of the patients in relation to survival are shown in Table 4. Patients who survived had a lower ISS (*p* = 0.011) and lower rates of pre-hospital and hospital hemodynamic instability (*p* = 0.031 and *p* = 0.003, respectively). Fifty-five of the 56 patients who underwent an immediate thoracotomy had obtainable SOL after injury; 50 of the 55 still had SOL at the ED. One patient who lost SOL at the ED did not receive resuscitative interventions at the ED, but underwent an ET instead of an EDT. All six patients who lost SOL died. Patients who died had a higher prevalence of concomitant abdominal injury (Table 4). The finding of peritoneal and retroperitoneal fluid during the operation, suggesting the existence of additional abdominal trauma, also coincided with a higher mortality rate (*p* = 0.009 and *p* = 0.036, respectively). Conclusively, patients who died showed a higher rate of transected aorta or vena cava (*p* = 0.018). Suspected pericardial tamponade, on the other hand, had a more favorable outcome (*p* = 0.003).

## Discussion

Nowadays, an EDT or an ET is performed in emergency situations following life-threatening thoracic—especially penetrating—trauma [8, 24, 25]. Guidelines for the treatment of thoracic injuries were established after World War II, and were derived originally from military experience [16]. In 2001, the National Association of Emergency Physicians and the American College of Surgeons composed a series of guidelines [3]. An EDT is recommended in patients who have sustained penetrating thoracic (cardiac) injuries and arrive at the trauma center after short on-scene and transportation times with witnessed or objectively measured SOL. However, physiological predictors of outcome, definitions of SOL, and the method used to identify patients in whom an immediate thoracotomy can be life-saving remain subjects for debate [3, 8, 14, 26–29]. Furthermore, outcome data from high-incidence regions like the US and South Africa may not be generalizable to the European population. Therefore, in this article, we have described our ten years of experience with immediate thoracotomies in a European level I trauma center.

The survival rate after an EDT published by the American College of Surgeons Committee on Trauma (ACSCOT) was only 11.2 %, among whom approximately 15 % survived with neurological impairment [3]. In our cohort, three out of 12 patients survived until discharge following an EDT; all were discharged without neurological impairment. Our survival rates compare favorably to other European studies in which mortality rates after EDT or ET of up to 100 % were found [15]. The most promising European experience so far has been the Glasgow series [30], with a 32 % survival rate (i.e., eight out of 25 patients survived) following immediate thoracotomy. Our overall survival rate of 64 % (36 out of 56 patients) is twice as high. The survival rate in the Glasgow series following an EDT was 6 %, which is much lower than the observed survival rate of 25 % in our level I trauma center. In order to determine if our favorable outcomes could be partly caused by overtreatment, preoperative indications were compared with the operative findings. When analyzing the EDTs, it seemed that the three patients who survived an EDT initially manifested with radiographic signs of a large hemothorax, shock, and signs of a pericardial-tamponade-like pericardial effusion on ultrasound or CTA. Consecutive operative findings were: laceration of the lung parenchyma, myocardial rupture, and laceration of the lung parenchyma. All patients were in severe shock (i.e., SBP <60 mmHg) and unresponsive to resuscitation. These patients could not have been transported to the OR for surgical treatment, and thus underwent an EDT. The abovementioned findings suggest that the decision to perform an EDT in these cases was adequate. Moreover, indications were in accordance with the ATLS and ERC guidelines [19, 31]. Based on our study findings, we are confident that the standard of care in combination with the developed treatment algorithm as shown in Fig. 1 allows us to achieve a relatively favorable outcome. Nevertheless, deciding on whether or not to perform an immediate thoracotomy remains a challenge.

Several indications, including specific physical parameters, were proven to be associated with a favorable outcome [3, 5, 14, 17, 19, 32, 33]. In our study, certain indications such as the presence of SOL, suspected traumatic pericardial tamponade, or the presence of concomitant abdominal injury were found to have a significant influence on the outcome after EDT or ET.

Loss of SOL is an important variable describing a patient’s physical condition that presented more often in the patients who died. Nevertheless, controversy exists over when and which SOL are related to a better outcome [34]. An immediate thoracotomy is believed to be beneficial in patients who arrive with vital signs at the ED or in those with a witnessed loss of SOL, not in those who are already showing no SOL before the (helicopter) emergency medical services have arrived at the scene of injury [3]. In our cohort, obtainable SOL were present in all 36 survivors. Survivors, however, did not show all possible SOL; two lost their pupillary response after injury, one suffered a prehospital asystole that persisted until arrival at the ED, and one showed a loss of SOL during the EDT. Seamon et al. reported similar findings and suggested that EDT can have a favorable outcome as long as one or more SOL are present at the scene of injury. Moreover, the moment in time when the SOL were observed seemed to affect the outcome [32]. All five patients who demonstrated recordable SOL at the incident scene but lost all SOL at or during transportation to the ED died in our study. Several authors support the theory that a witnessed loss of SOL is one of the indications to perform an immediate thoracotomy [3, 35]; however, our data proved that a poor outcome followed a witnessed loss of SOL. Considering this outcome, it was noted by Hall et al. that current recommendations to perform an immediate thoracotomy might be a little optimistic. They proposed that they are mainly based on the outcomes of the more specialized and experienced institutions, where immediate thoracotomies are performed more routinely [35]. Another option for improving the survival of patients with a witnessed loss of SOL might be a pre-hospital thoracotomy following the indications mentioned by Coats et al. [36]. Altogether, loss of SOL as an indication for an immediate thoracotomy deserves extra observation in the future, focusing in particular on low-incidence regions. Concomitant abdominal injury was found to be more prominent among the patients who died, which is in agreement with several studies from high-incidence regions [5, 6, 37, 38]. Mortality rates in our study were higher in patients receiving both a thoracotomy and a laparotomy. Negative laparotomy rates of up of 30 % were seen in cases with thoracoabdominal injuries [39, 40], with complication rates of 2.5–41 % [41]. Both findings reflect the importance of a reliable diagnostic approach for thoracoabdominal injuries. Further research in this area is desired, since most studies describe diagnostic imaging following blunt, not penetrating, trauma [42–45].

As for cardiac injury, the ACSCOT guidelines support the use of an EDT in hemodynamically unstable patients or patients with a witnessed loss of SOL in whom a pericardial tamponade is suspected. The ACSCOT guidelines also state that an EDT can be used as a diagnostic tool for discriminating cardiac from noncardiac thoracic injury [3]. In our center, clinical or CXR suspicion of pericardial tamponade (PT) is treated according to our algorithm (Fig. 1). Ultrasound-confirmed pericardial effusion (>8 mm) in patients with an SBP of <60 mmHg prompts immediate EDT. In patients with an SBP of >60 mmHg who undergo an ET for additional injuries, the pericardium is opened to assess the myocardium for injuries. In hemodynamically stable patients, the pericardium is inspected via the subxiphoid pericardial window (SPW) technique, as described by Arom et al. [46]. In cases with gross blood drainage from the pericardial sac, the procedure is converted into a sternotomy to treat the injuries to the heart. If only serosanguinolent fluid is encountered, a drain is placed in the pericardial sac until the output is less than 50 mL over 12 h, as advocated by Navsaria et al. [47]. In our cohort, patients with a suspected traumatic pericardial tamponade were more abundant among the survivors, suggesting a more favorable outcome [36, 48, 49]. Since outcome data from the high-incidence regions may not be generalizable to low-volume areas such as most European countries, further research from low-incidence regions is needed. Despite a lower occurrence of penetrating thoracic injuries, we were able to show that performing immediate thoracotomy in a level I trauma center in a lower-incidence region can produce similar outcomes to those seen in high-incidence regions. However, since immediate thoracotomies are not part of the daily routine of most trauma centers in these low-incidence regions, cooperation between different European hospitals could help to improve penetrating trauma research in the future. In addition, training programs in high-volume centers, in combination with recurrent surgical technique training on cadavers, may contribute to better outcomes.
